# Body image and health-related behaviors among fitspirit participants

**DOI:** 10.1186/s12889-022-14131-7

**Published:** 2022-09-17

**Authors:** Manon Bordeleau, Jo-Anne Gilbert, Natalie Alméras, Johana Monthuy-Blanc, Joël Gagnon, Marie-Ève Mathieu, Vicky Drapeau

**Affiliations:** 1 Centre de recherche de l’Institut universitaire de cardiologie et de pneumologie de Québec, Université Laval (IUCPQ-UL), Québec City, Québec Canada; 2 Department of Physical Education, Université Laval, Québec Québec City, Canada; 3 Centre de recherche interuniversitaire sur la formation et profession enseignante (CRIFPE), Université de Montréal, Montréal, Québec Canada; 4 Centre Nutrition, santé et société (NUTRISS), Institut sur la nutrition et les aliments fonctionnels (INAF), Université Laval, Québec City, Québec Canada; 5 École de kinésiologie et des sciences de l’activité physique, Université de Montréal, Montréal, Québec Canada; 6 Sainte-Justine University Health Center, Université de Montréal, Montréal, Québec Canada; 7 Department of Kinesiology, Faculty of Medicine, Université Laval, Québec City, Québec Canada; 8 GR2TCA-Loricorps, Groupe de Recherche Transdisciplinaire des Troubles du Comportement Alimentaire, Department of Education Sciences, Université du Québec à Trois-Rivières, Québec, Canada; 9 Centre de recherche de l’Institut universitaire en santé mentale de Montréal, Québec Montréal, Canada

**Keywords:** Body size perception, Body size dissatisfaction, Healthy habits, Physical activity, Adolescent girls, Health behaviors, Self-perception, Obesity

## Abstract

**Background:**

Body image variables such as body size perception (BSP) and body size dissatisfaction (BSD) can influence health-related behaviors. However, few studies have investigated these body image variables in adolescent girls participating in a physical activity intervention. Therefore, the study objectives were to examine the 1) associations between health-related behaviors (physical activity, screen-time, eating habits and sleep duration) and BSP and BSD among girls participating in FitSpirit, a physical activity intervention for girls; and 2) influence of weight control practices on the association between health-related behaviors and BSP and BSD.

**Methods:**

This cross-sectional study assessed a sample of 545 adolescent girls (mean age: 15.0±1.5 years) from 240 schools. Body mass index, health-related behaviors, perceived actual body size and desired body size variables were self-reported and collected via an online questionnaire at the end of the FitSpirit intervention. A negative BSP score [perceived actual body size – calculated BMI *z*-score] indicates an underestimation of body size. A positive BSD score [perceived actual body size – desired body size] indicates a desire to reduce body size. A multiple linear regression analysis examined the effects of age, zBMI and health behaviors on BSP and BSD. A second multiple linear regression analysis examined the independent associations between BSP and BSD by weight control practice. The linear relationships between BSP and BSD were evaluated with Pearson’s correlations.

**Results:**

Underestimation and dissatisfaction of body size are more prevalent in participants living with overweight/obesity. Screen-time and sleep duration were independently associated with BSP score (Beta=0.02; *P*<0.05 and Beta=-0.07; *P*<0.05, respectively), whereas only screen-time was associated with the BSD score (Beta=0.07; *P*<0.001). Physical activity was independently associated with the BSP score only in participants trying to control (maintain) their weight (Beta=-0.18; *P*<0.05).

**Conclusions:**

Body size overestimation and dissatisfaction are associated with health-related behaviors, specifically with more screen-time and less optimal sleeping habits. Physical activity level does not appear to be associated with body image in girls engaged in a physical activity intervention and who want to lose or gain weight. Health promotion interventions could include screen-time and sleep components as they may influence body image.

## Background

Research has shown that regular physical activity (PA) and low sedentary time are positively associated with physical, social and mental health [1, 2]. Despite these known benefits, the latest evidence suggests that PA levels decline drastically during the transition from childhood to adolescence, especially in girls, while sedentary time increases considerably [3]. In Canada, only 14% of adolescent girls meet the daily 60 min of moderate-to-vigorous PA recommendations [4] which can be a critical determinant of obesity [5]. Compared to normal weight girls, adolescent girls living with overweight/obesity (OW/OB) are more likely to engage in unhealthy weight control practices [6]. Past research has shown that there is a bidirectional association between body image variables, such as body size perception (BSP) and body size dissatisfaction (BSD), and obesity-related behaviors, including an unhealthy lifestyle and weight control practices [6–8].

Several studies suggest that adolescent girls living with OW/OB are more likely to underestimate their body size, which can impact the adoption of certain health behaviors [7, 9]. For example, a previous study conducted in Québec found that 90% of adolescent girls living with OW/OB failed to identify their size compared to 55% of underweight and 13% of those normal weight [10]. Past research has shown that misperception can be an antecedent to healthy and unhealthy behaviors [7]. Body size overestimation is associated with a greater screen-time [11], higher vegetable consumption [12] and unhealthy weight control practices [7]. On the other hand, adolescents who underestimate their body size have a lower level of PA [13] and are less likely to adopt unhealthy weight control practices [14].

There is evidence that body size dissatisfaction is highly prevalent in girls, especially those living with OW/OB [15]. A study in Québec demonstrated that, in adolescent girls, the desire to reduce body size was more present than the desire to increase body size (47% vs. 11%, respectively) [10]. Furthermore, 81% of adolescent girls living with OW/OB reported being dissatisfied with their body size compared to 45% of underweight and 52% of normal weight [10]. The aspiration to reduce body size has been associated with obesity-related behaviors such as poor sleep [16] and low consumption of fruits and vegetables [17]. BSD has also been shown to behave as both a motivation and barrier to PA [18, 19]. For example, it has been reported that individuals with a high BSD may avoid opportunities to exhibit their bodies in a public setting or be exposed to weight criticism during PA [18]. In contrast, it has been shown that some adolescent girls may engage in PA to improve or maintain a desired physical appearance [19]. Body size satisfaction may positively influence health behaviors in this population and protect against harmful weight control practices [20].

Adolescent girls were identified as a target population for several school-based interventions that aimed to increase the practice of PA and reduce screen-time [21–23]. Over the past 20 years, many health-promotion initiatives have been implemented to encourage young girls to adopt a healthy and active lifestyle [22, 24]. Through these various interventions, evidence suggests that "girls-only" school-based interventions, such as FitSpirit [25], may be successful approaches to increase PA [21, 26]. However, there are a lack of studies examining the association between body image variables, such as BSP and BSD, and health behaviors in adolescents participating in a "girls-only" school-based intervention.

Examining the association between body image variables and health behaviors is important to better understand this relation and intervene more specifically in “girls-only” school-based interventions. In this context, the first objective of this study was to determine whether health-related behaviors [PA, screen-time, sleep duration and vegetable/fruit consumption], are independently associated with BSP and BSD among FitSpirit participants. The second objective was to investigate the association between BSP and BSD according to weight control practices. Finally, we hypothesized that misperception and dissatisfaction would vary with weight control practices and weight status.

## Methods

### Participants

The participants of this study took part in FitSpirit, a girls-only school-based intervention designed by a nonprofit organization (FitSpirit.ca). Since 2007, FitSpirit has helped schools organize activities and events for adolescent girls and engages approximately 12 000 participants yearly [27]. FitSpirit aims to raise adolescent girls’ motivation and enjoyment of regular PA by offering them different opportunities to be physically active and thus, promote the adoption of an active lifestyle in the short and long-term [25]. The program offers 4 types of activities to the participants, and each school decides which combination of them they put forward every year: (1) motivational conferences given by inspiring women, (2) weekly physical activity sessions, (3) a running program, and (4) a celebration day at the end of the school year to explore new activities in a non-competitive environment. School group activities are organized and led by a school staff member (FitSpirit leader). The intervention is generally carried out from February to May, but could start earlier. Approximately 250 high schools partner with FitSpirit across two Canadian provinces (Québec and Ontario) every year. The detailed design of this program is described by Leduc et al. [25].

**Fig. 1:**
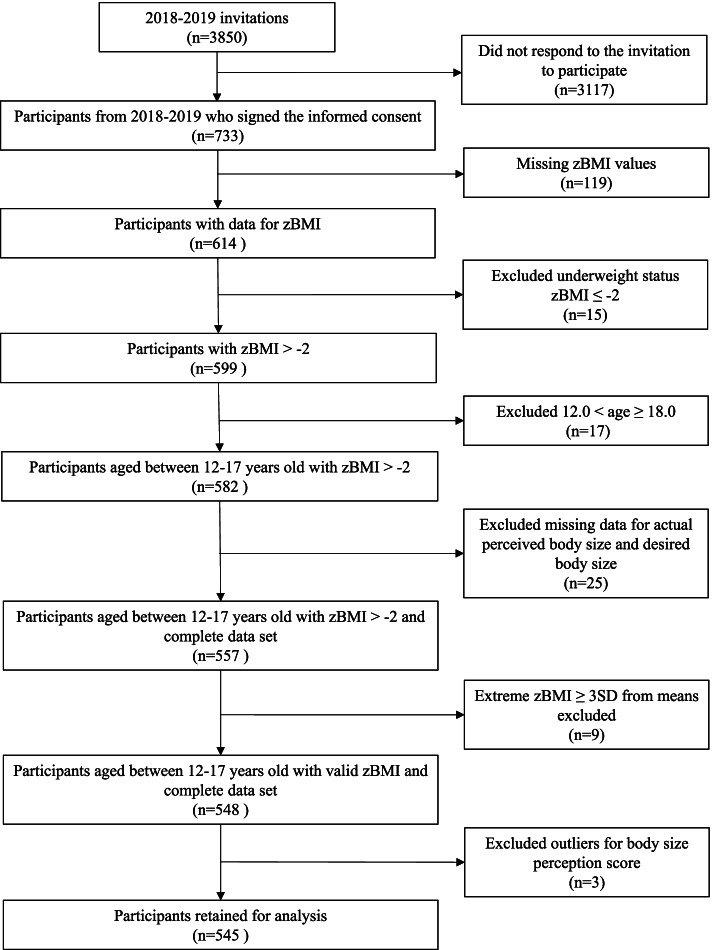
Flowchart of the study selection process

In the Spring of 2018 and 2019, FitSpirit participants from each school engage in the FitSpirit program were invited to participate in the evaluation program [25]. Recruitment was done through the FitSpirit leader who directly invited the girls involved in the FitSpirit program to participate in the program evaluation. FitSpirit participants interested by this evaluation signed an informed consent (*n*=733). At the end of the school year, which corresponds to the end of the intervention, FitSpirit sent an email inviting these participants to complete an online questionnaire on their experience with the program in French and English. Participants were selected for the present study based on the following criteria in order to have a more heterogeneous sample: aged between 12 and 18 years, normal weight or above for age, and have completed data for BMI, BSP and BSD. Fig. 1 depicts a flowchart of the selected research participants.

### Anthropometric measures

Body mass index (BMI) was calculated from self-reported height and weight (kg/m^2^) and converted to a standardized z-score using age- and sex-specific 2007 WHO growth reference charts for 5-19 years [28]. Scores were used to classify the weight status of adolescents as underweight (zBMI < -2 standard deviation), normal weight (1 > zBMI ≥ -2), overweight (2 > zBMI ≥ 1) or obese (zBMI ≥ 2) [28].

### Health-related behaviors

Self-reported PA was measured using the modified version of the short form of the International Physical Activity Questionnaire (IPAQ-SF) [29]. First, girls were asked about the total minutes spent on active travel and active leisure in the last seven-day period. Total PA time was calculated as the sum of these two results and then converted to mean minutes per day. Subsequently, screen-time was used as a surrogate measure of sedentary time. The girls self-reported the average time they spent on a typical day, watching TV, playing computer and video games, using a computer (also for homework), cellphone, etc. Thereafter, screen-time was converted to mean hours per day. Next, sleep duration was assessed with a single question asking girls to estimate, on average, how many hours they sleep in 24 hours. Consumption of vegetables and fruits was assessed using adapted questions from a validated self-reported survey [30]. Adolescent girls were asked to estimate how many days per week (0-7) and how many servings per day they consumed vegetables and fruits (not counting juice). In the 2019 form, girls were asked how many FitSpirit activity sessions they have participated during the 4 to 5 months of FitSpirit program 2019.

Current weight control practice was assessed using the questions taken from the Project EAT (Eating and Activity over Time), which was specifically designed to explore eating and weight-related issues among diverse young people across the life course [31]. Participants were asked the following question: Are you currently trying to . . . (1) lose weight or be thinner, (2) control (maintain) weight, (3) gain weight/muscles or be more defined, or (4) I am not trying to do anything about my weight.

### Measurements of perceived actual body size and desired body size

BSP and BSD were assessed using the Collins Figure Rating Scale [32], consisting of a series of seven-line drawing figures, ranging from extremely thin to obese with a middle figure representing a normal body size. Girls were asked to select the silhouette they believed was most similar to their own (perceived actual body size) as well as the body figure they most desired to look like (desired body size). We standardized the Collins figure scale for the perceived and desired body size as described by Maximova et *al*. [33] so that each figure was assigned a corresponding *z*-score (-3, -2, -1, 0, 1, 2, 3). Because the silhouettes are correlated with BMI percentiles [34], *z*-scores of -1, 0 and 1 represent normal body weight, while *z*-scores of 2 and 3 standard deviations above and below the mean have been associated with the overweight/obese (OW/OB) and underweight categories, respectively.

Following this procedure, the BSP score was calculated as the discrepancy between the perceived actual body size and actual body size (zBMI). According to the method described by Bordeleau et *al*. [35] using the BSP score, girls were classified into three groups: Underestimators (BSP score < -0.5); Accurate estimators (-0.5 ≤ BSP score ≤ 0.5); and Overestimators (BSP score > 0.5). Similarly, subtracting the desired body size from the perceived body size yielded the BSD score. According to the results obtained, the girls were also classified into three groups: desire to reduce body size (BSD score > 0.5); satisfied (-0.5 ≤ BSD score ≤ 0.5); and desire to increase body size (BSD score < -0.5).

### Statistical analyses

Student’s t-test was used to explore the mean of continuous variables, e.g., PA, screen-time, sleep duration and fruit and vegetable consumption, and difference between weight status subgroups. Chi-squared (χ2) tests were used to examine differences in the prevalence of each categorical variable for BSP (accuracy, underestimation and overestimation) and BSD (satisfaction, desire to reduce body size and desire to increase body size). Multiple linear regression models were analyzed to examine the effects of age, zBMI and health behaviors on BSP and BSD scores. Models with the lowest Akaike information criterion (AIC) were chosen. Girls were then divided into four groups according to their current weight control practice: Trying to lose weight or be thinner, trying to control (maintain) weight, trying to gain weight/muscles or be more defined, and not trying to do anything. A second multiple linear regression analysis examined the association between BSP and BSD by weight control practice. The linear relationship between BSP and BSD according to weight control practices was evaluated with Pearson’s correlations. Finally, Pearson’s correlation analyses investigated the associations between BSP and BSD adjusted for age. Statistical significance was considered at *P*-value ≤ 0.05. All analyses were performed with SAS OnDemand for Academics (Cary, NC, USA).

## Results

### Participant characteristics

The mean age for the 545 adolescent girls was 15.0 ± 1.5 years. For the total sample, about 77% were considered normal weight, while 16% were classified as overweight and 7% obese. The descriptive statistics of adolescents’ characteristics are presented by weight status in Table 1. In both the normal weight and OW/OB subgroups, mean perceived actual body size was significantly smaller (*p*<0.05) than self-reported zBMI (-0.5 ± 1.0 vs. -0.2 ± 0.7 and 0.9 ± 0.8 vs. 1.7 ± 0.7, respectively). Furthermore, mean desired body size was smaller than BSP score in the normal weight subgroup, but higher in the OW/OB subgroup (Table 1). Among the health behaviors, sleep duration was the only one for which there was a significant difference between the weight status subgroups.

**Table 1:** Characteristics of participants classified by body weight status

	**Normal weight** **(*n*=422)**	**Overweight/obese** **(*n*=123)**	***p*-value**
**Body image variables**			
Age (y)	15.0 ± 1.5	15.2 ± 1.4	0.10
	(12.0, 17.8)	(12.0, 17.8)	
zBMI	-0.2 ± 0.7	1.7 ± 0.7	< 0.001
	(-1.88, 0.98)	(1.00, 3.98)	
Perceived actual body size	-0.5 ± 1.0	0.9 ± 0.8	< 0.001
	(-3.0, 3.0)	(-1.0, 3.0)	
Desired body size	-0.9 ± 0.9	-0.7 ± 0.9	< 0.05
	(-3.0, 3.0)	(-3.0, 1.0)	
Body size perception score	-0.3 ± 0.9	-0.9 ± 0.9	< 0.001
	(-2.9, 3.4)	(-4.0, 1.0)	
Body size dissatisfaction score	0.4 ± 1.1	1.5 ± 1.0	< 0.001
	(-5.0, 6.0)	(-1.0, 6.0)	
**Health-related behaviors**			
Physical activity (h/day)	1.2 ± 1.0	1.2 ± 1.1	0.83
	(0.0, 7.1)	(0.0, 6.2)	
Screen time (h/day)	3.4 ± 2.0	3.4 ± 1.9	0.92
	(0.4, 10.1)	(0.6, 10.0)	
Sleep duration (h/day)	8.1 ± 1.4	7.8 ± 1.5	< 0.05
	(3.0, 13.0)	(3.2, 12.0)	
Fruits/Vegetables (servings/day)	3.6 ± 2.1	3.2 ± 2.0	0.10
	(0.1, 8.0)	(0.3, 8.0)	

Data are presented as non-adjusted means ± SD (min-max).

### Body size perception and body size dissatisfaction

**Fig. 2:**
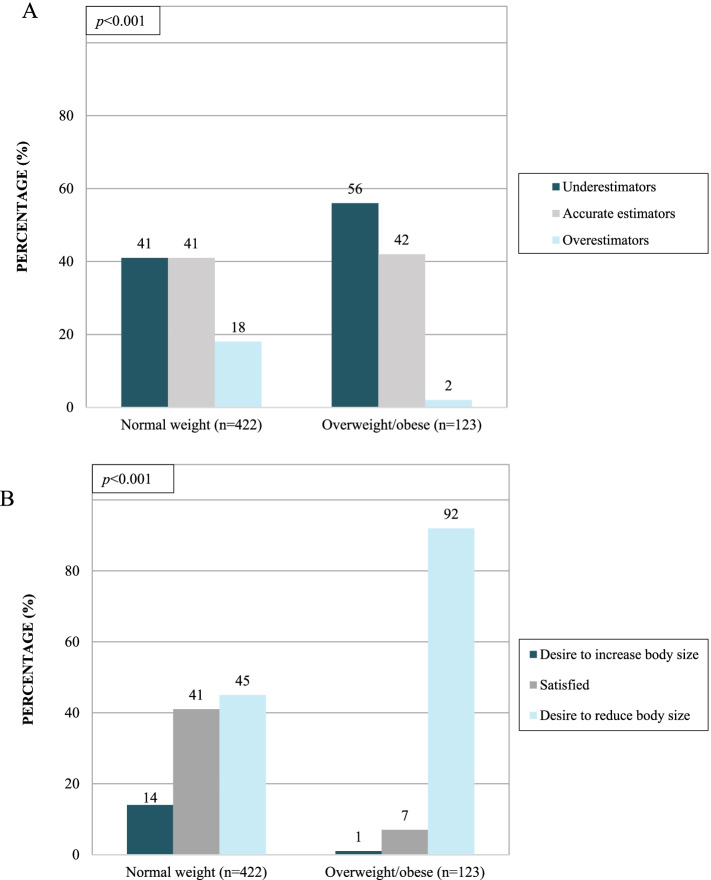
Prevalence of categorization of body size perception (accuracy, underestimation and overestimation) and body size dissatisfaction (satisfaction, desire to reduce body size and desire to increase body size) by weight status (normal weight and overweight/obese) in girls (*N*=545)

Mean BSP score was lower in the OW/OB subgroup, and BSD score was smaller in the normal weight subgroup (Table 1). Moreover, 41% of the girls accurately self-evaluated their body size (“Accurate estimators”), 45% thought they were thinner (“Underestimators”) and 14% thought they were bigger (“Overestimators”) than they were (zBMI). Fig. 2A shows that the prevalence of categorization of BSP was different according to weight status. Underestimation was more prevalent among girls living with OW/OB compared to the normal weight group (56% vs. 41% respectively). Regarding BSD, 34% of the total sample reported being satisfied with their body size, while 55% expressed the desire to reduce their body size and 11% to increase their body size. Fig. 2B shows that 45% of normal-weight girls wanted to reduce their body size while this proportion rose to 92% among the OW/OB subgroup.

### Associations between Body size perception, body size dissatisfaction and health-related behaviors

Table 2 presents the effect of age, zBMI and health behaviors [PA, screen-time, sleep duration and vegetable/fruit intake] in BSP and BSD scores. Results revealed that screen-time and sleep duration were independently associated with BSP score (Beta = 0.05, *p*<0.05; Beta = -0.07, *p*<0.05, respectively). Screen-time was the only health behavior associated with the BSD score (Beta = 0.07, *p*<0.001).

**Table 2:** Contribution of health behaviors on body size perception and dissatisfaction scores

		**Body size perception score**						**Body size dissatisfaction**			
**Variables**	R^2^	Partial R^2^	Beta	*p*-value	95% CI	R^2^	Partial R^2^		Beta	*p*-value	95% CI
	0.19					0.32					
Age (y)		0.03	0.07	0.004	[0.02, 0.13]		0.00		0.05	0.09	[-0.01, 0.10]
zBMI		0.13	-0.33	< 0.001	[-0.39, -0.26]		0.30		0.58	< 0.001	[0.51, 0.66]
Physical activity (h/day)		---	---	---	---		---		---	---	---
Screen-time (h/day)		0.02	0.05	< 0.05	[0.02, 0.09]		0.02		0.07	< 0.001	[0.03, 0.11]
Sleep duration (h/day)		0.01	-0.07	< 0.05	[-0.12, -0.01]		---		---	---	---
Fruits/vegetables (servings/day)		---	---	---	---				---	---	---

*CI* confidence intervals;

---: Variable not retained in the model with the lowest Akaike’s Information Criterion-corrected (AIC).

AIC for BSP score = -176.74; AIC for BSD score = -83.59.

### Body image and health-related behaviors by weight control practices

In the present study, 56% of the OW/OB subgroup reported trying to lose weight or be thinner while this prevalence was 19% in the normal-weight subgroup. Furthermore, a higher percentage of girls with a normal weight were trying to control (maintain) their weight or gain weight/muscles or be more defined than girls living with OW/OB (31% vs. 22% and 20% vs. 10%; respectively).

As presented in Table 3, PA was only independently associated with BSP score among girls trying to control (maintain) their weight (Beta = -0.18, *p*<0.05). Thus, each hour of PA among this subgroup of girls was associated with a decrease of -0.18 in the BSP score. In contrast, each serving of fruit/vegetable was associated with a decrease of -0.1 in BSP score in adolescents who reported not engaging in weight control behaviors (Beta = -0.10, *p*<0.05). Regarding the other health-related behaviors, results vary by weight control behaviors. Screen-time was associated with an increase in BSP and BSD, each hour of screen-time was associated with a 0.12 increase in BSP and 0.06 increase in BSD among girls who have reported trying to control (maintain) their weight (Beta = 0.12, p<0.001 and Beta = 0.06, *p*<0.05). Screen-time was also independently associated with BSD score among girls who are not trying to do anything with their weight. Thus, each hour of screen-time was associated with an increase of 0.08 in the BSD score (Beta = 0.08, *p*<0.05). Finally, sleep duration was associated with a decrease of -0.12 in BSP score in adolescents who reported trying to lose weight (Beta = -0.12, *p*<0.05). Sleep duration is the only health-related behaviors independently associated with BSD score in girls trying to gain weight/muscles or be more defined. Thus, each hour of sleep among this subgroup was associated with an increase of 0.17 in the BSD score (Beta = 0.17, *p*<0.05).

**Table 3:** Contribution of health-related behaviors in body size perception and dissatisfaction scores by weight control behaviors

**Variables**	**Body size perception score**					**Body size dissatisfaction score**				
	**Trying to lose weight or be thinner (*n*=147)**									
	R^2^	Partial R^2^	Beta	*p*-value	95% CI	R^2^	Partial R^2^	Beta	*p*-value	95% CI
	0.29					0.11				
Age (y)		0.01	0.07	0.13	[-0.02, 0.17]		---	---	---	---
zBMI		0.23	-0.53	< 0.001	[-0.67, -0.38]		0.09	0.32	< 0.001	[0.15, 0.49]
Physical activity (h/day)		---	---	---	---		---	---	------	
Fruits/vegetables (servings/day)		---	---	---	---		---	---	---	---
Screen-time (h/day)		---	---	---	---		---	---	---	---
Sleep duration (h/day)		0.05	-0.12	0.007	[-0.21, -0.03]		0.02	-0.09	0.08	[-0.19, 0.01]
	**Trying to control (maintain) weight (n=157)**									
	0.38					0.13				
Age		---	---	---	---		---	---	---	---
zBMI		0.29	-0.51	< 0.001	[-0.64, -0.38]		0.10	0.24	< 0.001	[0.14, 0.35]
Physical activity (h/day)		0.03	-0.18	< 0.05	[-0.30, -0.05]		---	---	---	---
Fruits/vegetables (servings/day)		---	---	---	---		---	---	---	---
Screen-time (h/day)		0.06	0.12	< 0.001	[0.07, 0.18]		0.03	0.06	< 0.05	[0.01, 0.11]
Sleep duration (h/day)		---	---	---	---		---	---	---	---
	**Trying to gain weight/muscle or be more defined (n=98)**									
	0.04					0.46				
Age		---	---	---	---		---	---	---	---
zBMI		0.04	-0.18	< 0.05	[-0.35, -0.01]		0.42	0.87	< 0.001	[0.67, 1.07]
Physical activity (h/day)		---	---	---	---		---	---	---	---
Fruits/vegetables (servings/day)		---	---	---	---		---	---	---	---
Screen-time (h/day)		---	---	---	---		0.01	0.07	0.13	[-0.02, 0.17]
Sleep duration (h/day)		---	---	---	---		0.02	0.17	< 0.05	[0.02, 0.33]
	**Not trying to do anything (n=137)**									
	0.27					0.18				
Age		0.02	0.10	< 0.05	[0.00, 0.19]		---	---	---	---
zBMI		0.20	-0.45	< 0.001	[-0.60, -0.31]		0.16	0.37	< 0.001	[0.22, 0.51]
Physical activity (h/day)		---	---	---	---		---	---	---	---
Fruits/vegetables (servings/day)		0.05	-0.10	< 0.05	[-0.17, -0.03]		---	---	---	---
Screen-time (h/day)		---	---	---	---		0.03	0.08	< 0.05	[0.00, 0.15]
Sleep duration (h/day)		---	---	---	---		---	---	---	---

CI: confidence intervals;

---: Variable not retained in the model with the lowest Akaike’s Information Criterion-corrected (AIC);

^a ^Trying to lose weight or be thinner; AIC for BSP score = -75.09; AIC for BSD score = -65.43;

^b ^Trying to control (maintain) weight; AIC for BSP score = -72.74; AIC for BSD score = -124.94;

^c ^Trying gain weight/muscle or be more defined; AIC for BSP score = -36.36; AIC for BSD score = -6.57;

^d ^Not trying to do anything; AIC for BSP score = -50.07; AIC for BSD score = -51.22.

**Fig. 3:**
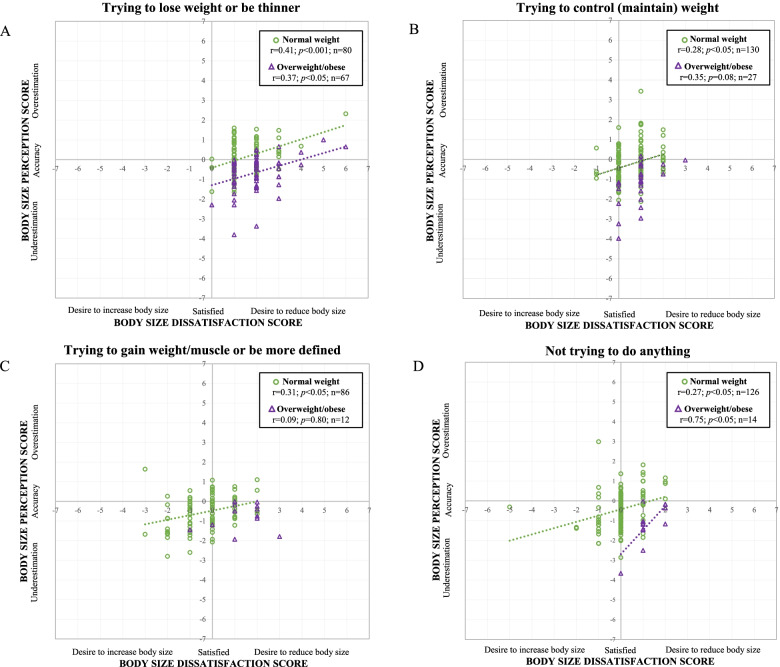
Trying to lose weight or be thinner (**A**), trying to control (maintain) weight (**B**), trying to gain weight/muscle or be more defined (**C**) and not trying to do anything (**D**)

Pearson’s correlations were conducted to examine the associations between BSP and BSD scores among participants reporting different weight control practices. Significant positive correlations between BSP and BSD among participants using different weight control practices were observed. However, the correlations by weight status subgroups were not all significant (Fig. 3). Fig. 3A shows significant positive correlations between BSP and BSD scores among girls trying to lose weight or be thinner in the normal and OW/OB subgroups. Moreover, the relationships between BSP and BSD were significantly different between the two subgroups (slope and intercept: *p*<0.001). In girls trying to control (maintain) their weight (Fig. 3B) and those who are trying to gain weight/muscles or be more defined (Fig. 3C), significant relationships were observed only in normal-weight girls. Finally, Fig. 3D shows significant positive correlations between BSP and BSD scores among girls who are not trying to do anything in the normal and obese subgroups. The relationships between BSP and BSD were significantly different between the two subgroups (slope and intercept: *p*<0.05).

## Discussion

Misperception and dissatisfaction of body size are common phenomena among adolescents [10]. Since BSP and BSD can be determinants of health behaviors and unhealthy weight control practices, it is important to question whether or not adolescent girls who engage in PA interventions have a positive body image. In response to these concerns, this study aimed to examine the associations between health-related behaviors, BSP and BSD in girls who participated in a PA promotion intervention and to document the degree to which behaviors are associated with the expression of BSP or BSD. These findings suggest that some health-related behaviors, i.e. screen-time, sleep patterns, are associated with overestimation and dissatisfaction, which may vary depending on weight control practices and weight status.

First, when compared by weight status subgroups the prevalence observed in a population of adolescents from the province of Québec, FitSpirit participants living with OW/OB appeared to assess their weight status more accurately (10% vs. 42%, respectively) while the normal-weight subgroup underestimated their body size (13% vs. 41%, respectively) [10]. Because of the cross-sectional nature of the present study, it is difficult to state whether participation in PA helps to improve accuracy in girls living with OW/OB, but some studies support this hypothesis [36]. One example of that is the study of Gesell et *al*. (2010), who found that a PA intervention among overweight children improves accuracy of body size perception [37]. A review also demonstrated that the increase in the tendency to underestimate body size may be explained by the “visual normalization theory” [38]. This theory suggests that one’s perception of his/her own body size depends on the size of the bodies this person is frequently exposed to in his/her environment [38]. In the present study, the proportion of adolescents living with OW/OB was higher than in the one carried out in Québec (23% vs. 19%, respectively) [10]. Hence, in FitSpirit, the higher exposure to heavier bodies may explain the greater underestimation of body size among normal weight participants and the more accurate perception among those living with OW/OB. In addition, this difference in prevalence between the two studies could also be explained by a potential selection bias. Adolescents involved in FitSpirit desired to improve their level of PA for different reasons, including reasons related to BSD.

Moreover, our results are in line with previous studies showing that adolescents living with OW/OB report body size dissatisfaction more frequently [10]. However, the present results revealed that girls living with OW/OB reported a higher proportion of the desire to reduce body size than another adolescent girl’s population from Québec (92% vs. 78%, respectively) [10]. This higher proportion could be explained by the context of the studies, including a possible selection bias induced by a higher interest toward PA in our study as well as a potential impact of the intervention itself. In addition, it has been shown that this type of intervention, exclusive to girls, could enhance the comparison of the body between the participants and may lead to a more frequent desire to reduce body size [39].

Regarding health behaviors, screen-time was positively associated with BSP and BSP. Thus, each hour of screen-time was associated with a significant increase of body size overestimation and a desire to reduce body size. Social media such as Instagram occupy an important proportion of screen-time in adolescents girls [40]. The users of these social media platforms are frequently exposed to appearance-highlighting content, including retouched images, leading to unrealistic body ideals [41, 42]. Adolescent girls who fail to meet these standards may view their body concerns increase, which can be related to body size overestimation and dissatisfaction [7, 43, 44]. In line with the meta-analysis results, higher screen-time may be associated with the desire to reduce body size [45].

Furthermore, research revealed that exposure to social media is linked to body dissatisfaction [45]. Higher social media usage increases opportunities for adolescent users to engage in social comparisons as they can compare themselves to their friends, family, influencers and celebrities [46, 47]. A recent study by Scully et *al.* (2020) suggests that body dissatisfaction increases significantly with time spent on social comparisons [48]. Furthermore, previous studies have shown that screen-time use can delay bedtime, reducing sleep duration in adolescents [49]. Correspondingly, the present study results showed that one hour less of sleep was associated with a body size overestimation. Thus, a possible relationship could exist between screen-time, sleep duration, and overestimation of body size.

Even though PA did not emerge as an independent variable associated with body image, past studies showed that PA could be indirectly related via its association with screen-time and sleep duration. Greater screen-time has been associated with a lower PA [50] while a higher PA has improved sleep duration [51, 52]. In addition, because the current study is cross-section, it is impossible to establish a causal relationship. Considering that the data were collected towards the end of the FitSpirit intervention, the study participants are those who have remained engaged until this moment and therefore, are more inclined to achieve a minimum of PA, which could have impacted the results. Nevertheless, the results of the study suggest that 53% of the participants meet the daily 60 min of moderate-to-vigorous PA recommendation which is higher than what has been found in similar populations [4]. Moreover, we do not have comparative data that would allow us to better analyze the impact of changes in PA on BSP and BSD during the FitSpirit intervention.

Another aim of the present study was to determine if the association between health behaviors with BSP and BSD differed by weight control practices. Results showed that fruit/vegetable consumption was associated with the underestimation of body size in those not trying to control (maintain) their weight. We also found that PA was only associated with underestimation in the subgroup currently trying to control (maintain) weight. This result is consistent with another study among primary school children that observed that girls who perceived themselves as overweight were favorable towards PA to control (maintain) weight [53]. Therefore, it is positive to note that PA level is not associated with BSP and BSD in the other weight control practices. Therefore, PA level is not related to body image variables in girls who are trying to lose weight or be thinner neither among girls trying to gain weight/muscles or be more defined engaged in a PA promotion intervention.

Given that BSP and BSD are influenced by body weight status and weight control practices, understanding the association between these BSP and BSD by body weight status and weight control practice may provide a further understanding of these associations. Accordingly, as observed in Fig. 3, we found significant correlations between BSP and BSD scores according to weight control practices and weight status. A relationship was present in all normal-weight subgroups regardless of the weight control practices. As observed in Fig. 3A, the correlation is similar between the two weight subgroups. However, in Fig. 3D, the correlation is much stronger in adolescent girls living with OW/OB. The positioning suggests that the greater underestimation is associated with body size satisfaction with this group. This result is in line with the study by Bordeleau et *al.* (2021) conducted among primary school children, where an underestimation in children living with obesity was associated with a lower desire to be thinner [35]. Thus, youth living with OW/OB who underestimate themselves protect themselves from a BSD and unhealthy weight control practices. Moreover, those who reported trying to control (maintain) their weight seemed to be satisfied with their body size and almost all girls living with OW/OB were underestimators. As mentioned, PA was associated with BSP only in girls trying to control (maintain) their weight. Considering that girls were assessed when they were already engaged in the intervention, they may have changed their habit towards PA which could have influenced their body size perception and satisfaction. Thus, although it is possible to note differences between perception and dissatisfaction by body weight status and weight control behaviors, studies are needed to further investigate their relationships and the association with health behaviors in longitudinal studies. Understanding adolescent girl’s BSP and its relationship with BSD is important to help develop interventions that encourage and cultivate a positive (vs. accurate) body image in adolescents of all sizes. Interventions that promote positive body image among adolescent girls should address or consider the role of some health-related behaviors such as screen-time and sleep duration. These interventions should also increase adolescent’s critical thinking around social media and the impact on their body size satisfaction.

### Strengths and limitations

The strengths of this study include the large number of participants and random selection process from the 240 participating schools. In addition, to our knowledge, no study evaluated the associations between BSP and BSD by weight control behaviors. Study limitations should also be considered when interpreting these findings. First, the cross-sectional design limits the analysis of temporal relationships between body image, health behaviors and weight control practices. Second, the intervention may have attracted girls interested in enhancing their PA level, which may not represent all adolescent girls. Third, FitSpirit is an intervention carried out by a school staff member with different levels of motivation [54], body image self-perceptions, and beliefs around weight control practices which may have influenced health-related behaviors in girls. Fourth, self-reported measurements were used in our study. Previous studies suggest that self-reported height is often overestimated and weight underestimated among adolescent girls [55]. This suggests that the BMI variable used in this study might be lower than measured BMI. Considering this point, it is possible that some girls have identified themselves with a larger figure than the one associated with their reported BMI which would increase the prevalence of overestimation. Nevertheless, overestimation is still trivial in our cohort (i.e., only 14% thought they were bigger “Overestimators”). Adolescents also tend to self-report a higher level of PA [56] which could have impacted the results obtained in the association between PA and body image variables. Finally, another limitation is related to the fact that the program does not have detailed information on program adherence. Of the 545 participants, 54% (*n*=297) answered the question about how many FitSpirit activities they attended during the program, which is usually from February to May. These activities include motivational conferences, weekly physical activity sessions, and other related events. An average of 12.0 ± 11.6 activities per participant were complete but it is important to consider that no information was provided on the nature, intensity, duration or when the sessions were performed which makes it difficult to assess real adherence to the program. Future research should consider adding social desirability questionnaire and a more objective measure of participation adherence such as using accelerometers to provide measures of PA. An additional limitation derives from the use of the Figural Rating Scale developed by Collins (1991) which considers only two dimensions of BSD (the desire to reduce or increase body size) and does not specifically refer to muscularity. Therefore, further studies should analyze the body size perception and dissatisfaction according to the aspect of muscularity.

## Conclusions

Findings from this study provide evidence that body size underestimation and dissatisfaction are common among girls participating in a PA promotion intervention, more specifically among adolescent girls living with OW/OB. The relationship between BSP and BSD scores depends on weight status and weight control practice. Screen-time and sleep duration were independently associated with body size perception whereas only screen-time was associated with body size dissatisfaction. PA was only associated with body size perception in girls trying to control (maintain) their weight, not in those trying to lose or gain body weight. This research is an important step in exploring the association between body image variables and health behaviors. Further work is needed to evaluate the impact of a PA intervention that includes other lifestyle habits such as screen-time and sleep duration. Interventions should also include an educational component to increase girls’ awareness of the negative impact of social media use on body image.

## Data Availability

The datasets used and/or analyzed during the current study are available from the first author upon reasonable request.

## Abbreviations

PA: Physical activity
BMI: Body mass index
BSP: Body size perception
BSD: Body size dissatisfaction
zBMI: BMI z-score
OW/OB: Overweight/obese
